# Femtosecond Laser and Big-Bubble Deep Anterior Lamellar Keratoplasty: A New Chance

**DOI:** 10.1155/2012/264590

**Published:** 2012-02-09

**Authors:** Luca Buzzonetti, Gianni Petrocelli, Paola Valente

**Affiliations:** Ophthalmology Department, Bambino Gesù Children's Hospital, Via Torre di Palidoro snc, Passoscuro, 00050 Rome, Italy

## Abstract

*Purpose*. To report the 12-month follow-up after big-bubble deep anterior lamellar keratoplasty (DALK) assisted by femtosecond laser that we have called IntraBubble. *Methods*. A 60 kHz IntraLase femtosecond laser (Abbott Medical Optics) firstly created a 30° angled intrastromal channel to insert the air injection cannula, 50 *μ* above the thinnest corneal site measured by Sirius Scheimpflug camera (CSO, Firenze, Italy), then performed a full lamellar cut 100 *μ* above the thinnest corneal point, and from the same corneal depth, created a mushroom incision. The lamella was removed, and the smooth cannula of Fogla was inserted into the stromal channel and air was injected to achieve a big bubble. The follow up is 12 months, and sutures were removed by the 10th postoperative month in all patients. Best Corrected Visual Acuity (BCVA), spherical equivalent and, by Sirius Scheimpflug camera (CSO, Firenze, Italy) keratometric astigmatism were evaluated. *Results*. All procedures were completed as DALK except 2 converted to PK because an inadvertent intraoperative macroperforation occurred. Mean postoperative BCVA was 0.8, mean spherical equivalent was -3.5 ± 1.7 D, and mean keratometric astigmatism was 4.8 ± 3.1 D. *Conclusion*. The femtosecond laser could standardize the big-bubble technique in DALK, reducing the risk of intraoperative complications and allowing good refractive outcomes.

## 1. Introduction

Recently, the femtosecond solid-state laser [1] was successfully used in several corneal surgical procedures. The technology consists of an infrared Nd:Glass laser beam focused at the desired corneal depth to induce optical breakdown (called photodisruption) without thermal or shockwave damage to the surrounding tissue, which particularly improves the reproducibility and outcomes of lamellar surgery [2–4]. The femtosecond laser technology facilitates flap creation in laser in situ keratomileusis, the creation of arcuate incisions and channels for intracorneal rings, and the preparation of donor and host tissue for the anterior, posterior, and penetrating keratoplasty (PK). Lastly it has been applied also to the cataract surgery.

Over time different surgical approaches have been proposed for Deep Anterior Lamellar Keratoplasty (DALK) [5–9], but the the big-bubble technique introduced by Anwar and Teichmann [10] in which air is injected into the deep stroma in an attempt to achieve a large air bubble between the Descemet's membrane and the stroma to facilitate pre-Descemet's plane dissection, probably at the moment results as the more common.

We proposed a variant of the big-bubble technique in DALK assisted by femtosecond laser which we have called IntraBubble [11] in whom the laser allows a pre-Descemet's plane lamellar dissection to a predefined corneal depth and the creation of a channel in the posterior stroma of the recipient, 50 *μ* above the corneal thinnest point, into which a smooth cannula for air injection can be introduced.

 In this paper, we report the 12-month follow-up results after big-bubble DALK assisted by femtosecond laser.

## 2. Materials and Methods

Thirty-five eyes of 35 patients (mean age  23 ± 6  (SD) years) affected by keratoconus were enrolled in the study.

All procedures were performed by one surgeon (L. Buzzonetti) under general anesthesia. Corneal trephination was performed using the IntraLase 60 KHz femtosecond laser (Abbott Medical Optics, Inc.) loaded with the IntraLase Enabled Keratoplasty (IEK) computer program that provides the creation of corneal lamellae of different shapes (mushroom, zigzag, Christmas tree, etc.). The IntraLase requires placement of a suction ring and an applanation lens. After obtaining a proper vacuum seal, we applied the applanation lens to provide a uniform reference plane for the laser. We used the same laser settings to achieve mushroom incisions for both recipient and donor. The donor cornea was removed from Optisol (Bausch & Lomb) storage, mounted on an artificial anterior chamber (Coronet Patient Artificial Anterior Chamber, Network Medical Products, Ltd.), and then treated under the 60 kHz femtosecond laser.

**Figure 1:**
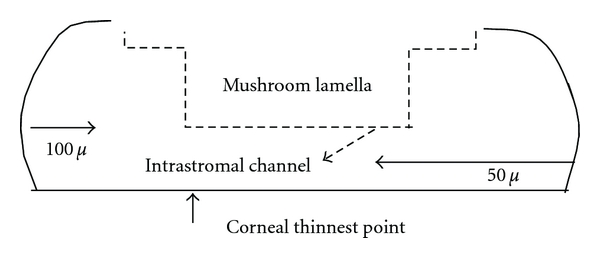
The dotted arrow shows the channel created in the recipient by the IntraLase femtosecond laser (30° angled, 25° arc length, and 6.0 mm diameter), 50 *μ* above the thinnest corneal site. The horizontal black dotted line shows the recipient lamellar cut 7.5 mm in diameter created 100 *μ* above the thinnest corneal site. The dotted profile shows the border line of corneal stroma after the mushroom lamella has been removed (anterior diameter of 8.0 mm and posterior diameter of 7.0 mm).

The center of the recipient was marked with a marking pen. In the recipient the laser treatment was planned in three consecutive steps as previously described by Buzzonetti et al. [11] (Figure 1).

**Figure 2:**
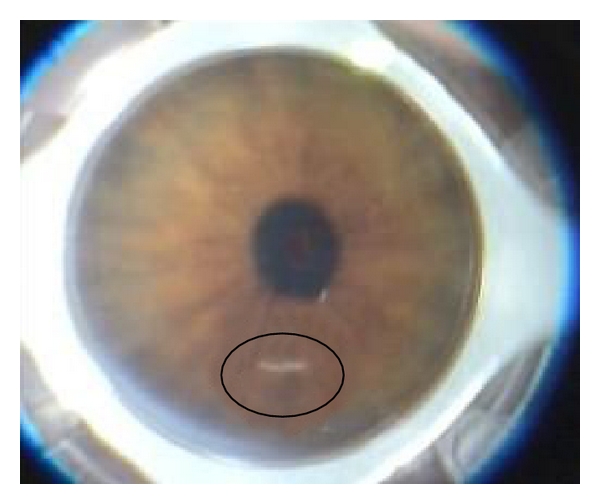
The femtosecond laser has created an intrastromal channel 50 *μ* above the thinnest point. The black circle shows it.

**Figure 3:**
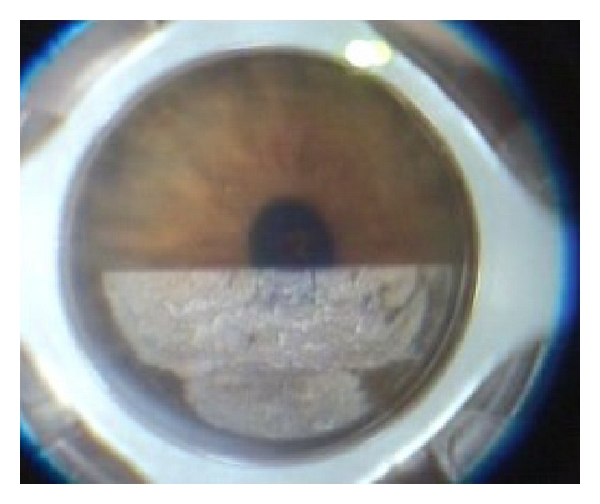
The femtosecond laser is creating the full lamellar cut 100 *μ* above the thinnest point.

1. The IntraLase performs an anterior side cut (30° angled, 25° arc length, and 6.0 mm diameter) 50 *μ* above the thinnest corneal site as measured by Sirius Scheimpflug camera (CSO, Firenze, Italy). The laser so creates an intrastromal channel (Figure 2).
2. A recipient lamellar cut 7.5 mm in diameter (Figure 3) is created by the IntraLase using a target thickness of 100 *μ* above the thinnest corneal site.
3. A mushroom lamella (anterior diameter of 8.0 mm and posterior diameter of 7.0 mm) is created by the IntraLase using the same target thickness of the lamellar cut (100 *μ* above the thinnest corneal site) because the lamellar cut depth has to correspond to the bottom side depth of the mushroom lamella.

**Figure 4:**
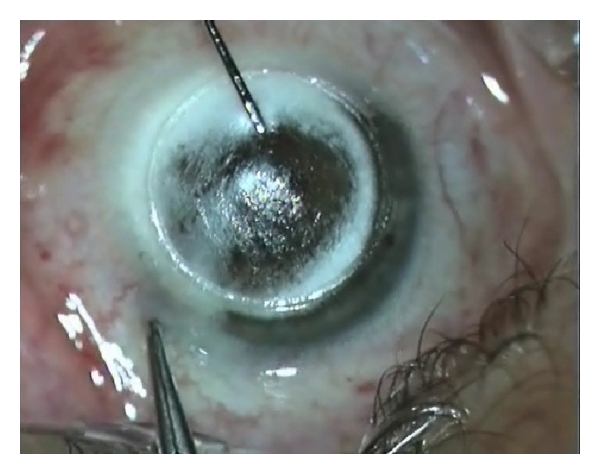
The big bubble has been achieved.

The recipient lamella was then removed, a Fogla-pointed dissector (Bausch & Lomb Storz Ophthalmic) was used to prolong the stromal channel created by the laser, too short to achieve the complete insertion of the cannula for air injection; then a Fogla 27 gauge air injection cannula (Bausch & Lomb Storz Ophthalmic), attached to a 5 mL syringe filled with air, was inserted into the residual stroma and slightly advanced into the channel. Air was then forcefully injected into the stroma to achieve the formation of a big-bubble (Figure 4). The surgery was then completed as the conventional big-bubble procedure: a peripheral paracentesis was performed, allowing some aqueous to escape to lower the intraocular pressure, and a small air bubble was injected into the anterior chamber to check the baring of Descemet's membrane. Then a 15° disposable knife was used to perforate the bubble and an ophthalmic viscosurgical device was injected to refill the space and protect the Descemet's membrane. The residual stroma was excised using corneal scissors and the smooth transparent Descemet's membrane appeared. Finally, the donor lamella, previously prepared by femtosecond laser, after the endothelium, was removed by sponge, was fitted into place, and sutured using 16 interrupted 10–0 monofilament nylon sutures.

The followup is 12 months, and sutures were removed by the 10th postoperative month in all patients. The following data were evaluated: Best Corrected Visual Acuity (BCVA), spherical equivalent and, by Sirius Scheimpflug camera (CSO, Firenze, Italy), keratometric astigmatism.

## 3. Results

All procedures were completed as DALK except 2 converted to PK because an inadvertent intraoperative macroperforation occurred. In 3 patients the bubble was not achieved, but the DALK was completed anyway by layer-by-layer stromal dissection. None other intra- or postoperative complications were observed. Twelve months after surgery mean postoperative BCVA was 0.8 (range: 0.5 to 1.0), mean spherical equivalent was −3.5 ± 1.7 D (range: −0.25 to 1.50 D), and mean keratometric astigmatism was 4.8 ± 3.1 D (range: 3.0 to 6.50 D).

## 4. Discussion

The DALK, alternative to PK in cases of a healthy endothelium, is the first choice for many surgeons that appreciate the advantages of extraocular surgery, the associated good visual outcome, and the absence of endothelial rejection due to the preservation of the recipient endothelium. However, technical difficulties, protracted operating times and the risk of corneal intraoperative perforation limit DALK's success.

 Like conventional big-bubble DALK, the IntraBubble technique decreases the risk of immune rejection. However, the creation of a channel for smooth air injection in the IntraBubble technique significantly reduces the risk of inadvertent perforation, the main complication of DALK. Moreover, the use of the femtosecond laser, allowing the maintenance of a predefined corneal depth very close to the endothelium, provides a good percentage of success in achieving the bubble (82%). In fact it has been reported [12] that if the needle is not advanced deep enough in the stroma, more difficult is the baring of Descemet's membrane.

In last months we were evaluating the application of the big-bubble DALK assisted by femtosecond laser to the pediatric patients [13]. Also if the number of patients treated until now is less and the followup is short, our early findings suggest that the IntraBubble technique could be applied also to the children in the attempt to decrease the high rejection percentage (the reported [14] prognosis for graft survival after pediatric PK is of approximately 50% at 1 year) and improve the refractive outcome because in my experience the residual astigmatism results are effectively lower after the keratoplasty is assisted by femtosecond laser, probably because of the lamellae geometry that provides a better surface contact between donor and recipient.

In conclusion we believe that IntraBubble could help to standardise the big bubble technique in DALK, reducing the learning curve for surgeons who approach the big-bubble technique as well as the risk of intraoperative complications, especially perforation which is the real risk with this procedure, and allowing good refractive outcomes for the good alignment of donor and recipient mushroom configuration, both in adults and children.
